# Study on the Protective Immunity Induced by Pseudotyped Baculovirus Expressing the E Protein of Tembusu Virus in Ducklings

**DOI:** 10.3390/genes14071316

**Published:** 2023-06-22

**Authors:** Zheng Ni, Tao Yun, Liu Chen, Weicheng Ye, Jionggang Hua, Yinchu Zhu, Guangqing Liu, Cun Zhang

**Affiliations:** 1 State Key Laboratory for Managing Biotic and Chemical Threats to the Quality and Safety of Agro-Products, Institute of Animal Husbandry and Veterinary Sciences, Zhejiang Academy of Agricultural Sciences, Hangzhou 310021, China; z_ni_hz@126.com (Z.N.); yt-t@163.com (T.Y.); haoliuzi@126.com (L.C.); ywc119@aliyun.com (W.Y.); huajg2008@126.com (J.H.); zhuyc111@163.com (Y.Z.); 2 Shanghai Veterinary Research Institute, Chinese Academy at Agricultural Sciences, Shanghai 200241, China

**Keywords:** DTMUV, E protein, protection, recombinant baculovirus, subunit vaccine

## Abstract

The Duck Tembusu virus (DTMUV), a pathogenic flavivirus, has been causing significant economic losses in the Chinese poultry industry since 2010. This virus can severely decrease egg production and inhibit the growth of laying ducks and ducklings. While many vaccines have been developed to prevent DTMUV infection, fresh outbreaks continue to occur, as few effective vaccines are available. The E glycoprotein of DTMUV is the primary target for inducing protective immunity in the natural host. Therefore, we conducted an investigation and successfully developed a recombinant baculovirus containing the DTMUV E gene. Ducklings were then vaccinated with the purified protein derived from this virus as a potential vaccine candidate. Our findings demonstrated that the E glycoprotein of DTMUV was highly expressed in Sf9 cells. The vaccination of ducklings with the recombinant baculovirus Bac-E resulted in the induction of strong humoral and cellular immune responses. Most significantly, we observed that the vaccine provided 100% protective immunity against lethal challenges with the DTMUV YY5 strain.

## 1. Introduction

Infectious disease outbreaks in ducks caused by the duck Tembusu virus (DTMUV) have been documented since April 2010 in all of China’s major duck-producing regions [1]. DTMUV is a single-stranded RNA virus belonging to a mosquito-borne *Flavivirus* of the *Ntaya* virus group, *Flavivirus* genus, and Flaviviridae family. It is a spherical and enveloped virus, approximately 40–60 nm in diameter [1,2,3]. The host profiles of DTMUV infection are very wide and can infect not only ducks but chickens, geese, pigeons and sparrows as well. Infection with DTMUV has shown serious systemic and neurological symptoms. Ducks with DTMUV infections frequently experience high fever, diarrhea and weight loss, as well as slowed growth and reduced egg production [3]. TMUV leads to encephalitis and neurological disorders in birds, causing high morbidity rates, whereas the fatality rate ranges from 10% to 30% [4,5,6]. This condition also affects the female reproductive system, resulting in a significant reduction of egg production in poultry farms [7]. Consequently, the development of a safe and effective vaccination to prevent this disease is crucial since the occurrence of DTMUV infection causes significant financial losses for the duck business [4,8,9,10]. DTMUV is a tiny, encapsulated, positive-stranded RNA virus with just one open reading frame (ORF) in its genome. Three structural proteins, capsid protein (C), pre-membrane (prM) and envelope (E) glycoprotein, as well as seven nonstructural (NS) proteins NS1, NS2A, NS2B, NS3, NS4A, NS4B and NS5, are encoded by the DTMUV genome [11,12,13]. The most crucial protective antigen of DTMUV among these proteins is the E protein, which, following virus infection, might trigger neutralizing antibodies [5,14,15]. As a result, the E protein plays a significant role in the immune response and may be a potential target for a DTMUV vaccine [15,16,17].

Clinically, vaccination is a successful method for preventing DTMUV infection. To preserve the duck industry, as well as to facilitate zero surveillance and zero monitoring, there is an immediate need for a DTMUV vaccine that is both more cost-effective and safe. Current DTMUV vaccines include inactivated vaccines and live-attenuated vaccines [17,18,19]. These vaccines have been successfully developed to control the occurrence and prevalence of DTMUV [19]. However, attenuated vaccines have the risk of virulence regression, and the safety of the inactivated vaccine is the main problem at present [20]. Therefore, the use of modern molecular biology technologies to develop new DTMUV vaccines has become a new research focus. To successfully express recombinant proteins for use in subunit vaccines, the baculovirus expression system has been widely used because the baculovirus expression system is a highly effective tool. This system has shown incredible promise in terms of immunogenicity, efficacy, cost and safety [21]. In addition to being completely risk free, it is capable of successfully inducing both humoral and cellular immune responses [22,23]. Most significantly, the apoptotic mechanism of these vaccines minimizes the possibility of viral DNA integrating into the genome of the host cell, which is advantageous for biosafety [23,24]. A subunit vaccine is an amazing method for preventing DTMUV infection because of all these benefits. 

In this study, we designed a recombinant baculovirus expressing the E glycoprotein of DTMUV and attempted to develop a novel subunit vaccine for E protein. Further research was conducted to investigate the efficacy of the pure recombinant E protein as a vaccine in terms of humoral and cell-mediated immune responses, in addition to protection against the DTMUV challenge in ducklings. Taken together, our findings suggest that the E protein could be a promising and practical vaccine candidate against DTMUV infection. 

## 2. Materials and Methods

### 2.1. Cell Materials and Animals

Using serum-free Sf-900TM II SFM (1) (Gibco, Grand Island, NY, USA), 100 U/mL penicillin, and 100 g/mL streptomycin, *Spodoptera frugiperda* 9 (Sf-9) cells were cultivated and maintained in monolayer cultures. The cultures were kept at 28 °C. In the Sf-9 cells, recombinant viruses were multiplied and titered. In the Sf-9 cells, proteins were also expressed. A virulent variant of the duck tembusu virus, strain YY5 (GenBank accession number JF270480), was discovered in 2013 during an outbreak in Zhejiang province, China and stored by our laboratory at the Zhejiang Academy of Agricultural Sciences (ZAAS). Disease Control YY5 was incubated and propagated on DF-1 cell monolayers, which were cultured in Dulbecco’s modified Eagle’s medium (DMEM; Invitrogen) with 2% (*v*/*v*) fetal bovine serum (FBS; Invitrogen) at 37 °C in a 5% CO_2_ incubator for 3 d. The infective dose in tissue culture (TCID50) and lethal dose in the chicken embryo (ELD50) were determined in our laboratory, which is 10^6^.^9^/mL and 10^3^.^1^/mL, respectively. The experimental ducklings were unimmunized Sheldrake ducklings (one-week-old, female) and were purchased from the Harbin Veterinary Research Institute in China. The ducklings were tested for the presence of the virus and seroprevalence before conducting the experiment.

### 2.2. Construction of the Recombinant Baculovirus rBac-E

The E gene sequence of the DTMUV strain YY5 (GenBank accession number JF270480) served as the basis for the design and synthesis of a specific primer pair by Qingke Biological Technology Co., Ltd. (Hangzhou, China) as follows: forward primer (P1- EcoR I) 5′-CCGGAATTCATGTTCAGCTGTCTGGGGATGC-3′ and reverse primer (P2- Xho I) 5′-CCCCTCGAGGGCATTGACATTTACTGCCAG-3′. The restriction sites are underlined.

The target E gene (1500 bp) was amplified by PCR using a protocol that started with initial denaturation at 94 °C for 5 min, followed by 30 cycles of 94 °C for 30 s, 55 °C for 30 s, and 72 °C for 90 s, and final extension at 72 °C for 10 min. The PCR product was purified and digested with EcoR I and Xho I restriction enzymes (Takara), then cloned into the shuttle vector pFastBac1 according to the supplier’s instructions. The resulting construct, pBac-E, was verified using DNA sequencing and enzyme digestion.

The recombinant baculovirus rBac-E was subsequently generated using the Bac-to-Bac System (Invitrogen, Carlsbad, CA, USA), following the manufacturer’s instructions. Site-specific transposition was used to integrate the construct pBac-E into the baculovirus genome of DH10 Bac (Invitrogen, Carlsbad, CA, USA). To construct a recombinant baculovirus, the recombinant bacmid was then transfected into Sf9 cells. Eventually, rBac-E was isolated using the plaque test three times after being further amplified by propagation in the Sf-9 cells. A wild-type baculovirus (wtBac) was used as a checkpoint. Using the Reed-Muench method, the viral titer TCID50 was calculated from the 8th generation of rBac-E.

### 2.3. Identification of DTMUV E Protein Expression in Sf-9 Cells

Sf9 cells were infected with rBac-E (MOI = 1) for 48 h, and DTMUV E protein expression was analyzed using an indirect immunofluorescence assay. After removing the supernatant, the cells were fixed with 100% acetone at −20 °C for 30 min. The cells were washed three times, then incubated with mouse anti-E polyclonal serum (at a dilution of 1:500) in a humid box at 37 °C for 60 min. Then, after three PBS washes, the cells were next incubated with FITC-conjugated goat anti-mouse IgG secondary antibody (1:1000, Beyotime Co., Beijing, China) at 37 °C for 60 min. The cells were washed three times, and a fluorescence microscope was used to observe the unique fluorescence of the infected cells. For Western blot analysis, the infected cells were collected, and cells infected with wild-type baculovirus (wtBac) were used as a negative control. In summary, the cells were collected and were lysed by the lysis buffer (Merck Co., Rahway, NJ, USA), and then lysates were separated by 10% SDS-PAGE and electro blotting to a nitrocellulose membrane (Bio-Rad, Hercules, CA, USA). The membrane was then treated with mouse anti-E polyclonal serum (at a dilution of 1:500) for 60 min at 37 °C after being blocked with 10% skim milk in PBST (0.05% Tween-20 in PBS) overnight at 4 °C. The secondary antibody was then the HRP-conjugated goat anti-mouse IgG (1:5000; Beyotime Co., Beijing, China). Lastly, 3,3-diaminobenzidine was used to detect immunoreactive E protein bands.

### 2.4. Experiments on Immunization and a Viral Challenge

Forty 1-week-old Sheldrake ducklings were confirmed to be free of anti-DTMUV antibodies. They were randomly selected and housed in isolators under positive pressure (ten ducklings in each group). Two study groups received subcutaneous injections of 50 μg cell precipitates that were resuspended with 1mL PBS and then ultrasonically broken, with recombinant rBac-E and wtBac in 200 μL of Freund’s adjuvant emulsion. The other two groups were immunized subcutaneously with either 200μL of the inactivated DTMUV vaccine (prepared by our laboratory) or PBS for use as a positive group and mock group, respectively. At 2-week intervals, ducklings from all vaccination groups received the same booster shot. Serum samples were collected at 0, 1, 2, 3 and 4 weeks post-primary immunization for the E protein specific antibodies against DTMUV and serum cytokine release assay. Two weeks after the second immunization, blood was collected for lymphocyte proliferation assay. All ducklings were intramuscularly challenged with 0.5 mL DTMUV strain YY5 containing 10^3^.^1^ ELD50/mL, four weeks after receiving their initial immunization. Following the challenge, all ducklings were monitored every day for 14 days.

### 2.5. Detection of Anti-DTMUV E Protein-Specific Antibodies

An indirect ELISA test was performed on serum samples from ducklings utilizing the recombinant DTMUV E protein generated in *Escherichia coli* BL21 (DE3) as an antigen. The PET 28a expression system (Novagen, Paris, France) was used to express the E protein in *E. coli* BL21 (DE3). After inducing the expression of the E protein in *E. coli*, the bacterial cells were lysed. The cleared lysate was then mixed with His resin and incubated at 4 °C for 30 min to allow binding of the E protein to the resin. The resin with the bound E protein was transferred to a purification column, and the liquid was allowed to flow out of the column under gravity. The gel was washed five times with wash buffer using the same volume as the cleared lysate to remove nonspecifically bound proteins. Subsequently, the elution buffer, in the same volume as the wash buffer, was added to the gel to elute the target protein. The eluted E protein was filtered using a 0.45 µM filter membrane and loaded onto a column packed with LPS resin to remove LPS from the E protein. In 0.1 M carbonate coating buffer (pH9.6), 96 wells of flat-bottomed plates (Corning Costar, Corning, NY, USA) were coated with recombinant E protein and incubated overnight at 4 °C. The plate was then washed three times with PBS containing 0.05% Tween-20 (PBST) and blocked with 5% BSA (Sigma-Aldrich, St Louis, MO, USA) in PBS for 1 h at room temperature (RT) on an orbital shaker. After washing an additional three times with PBST, the test serum samples diluted at 1:100 were added to the plates and incubated for 1 h at 37 °C. Control wells were set aside for the negative, positive and blank controls. The plate was washed with PBST 3 times and finally incubated with rabbit anti-duck IgG HRP conjugated secondary antibody (KPL, Gaithersburg, MD, USA) at a dilution of 1:2000 for 1 h at 37 °C. The assay reaction was developed using 1-Step Ultra TMB (Pierce, Waltham, MA, USA). Then, 2 M sulfuric acid was added for a stop solution. The relative quantification of E protein was followed by the addition of a Microliter Plate Reader from Bio-Rad, and absorbance was calculated at 450 nm.

### 2.6. Virus Neutralizing Antibody Test (VNT)

The DTMUV-specific neutralizing antibody titers from serum samples at weeks 0, 1, 2, 3 and 4 after primary immunization were detected using the VNT with DF-1 cells. Briefly, serum samples were heat-inactivated for 30 min at 56 °C to inactivate the complement. Then, 50 μL of two-fold serially diluted serum samples in DMEM was mixed with an equal volume of DMEM containing 200 TCID50 of YY5 strains, and the mixture was incubated at 37 °C for 1 h to neutralize the infectious viruses. Following this, these virus-serum mixtures were transferred to 96-well plates containing DF-1 cell monolayers and incubated for 5 days at 37 °C. Neutralization titers were calculated as the reciprocal of the highest serum dilution that completely inhibited viral replication in 50% of the wells.

### 2.7. Lymphocyte Proliferation Assay

Four weeks after receiving the first vaccination, a lymphocyte proliferation assay was performed. Briefly, a lymphocyte separation medium (Dakewe, Beijing, China) was used to extract peripheral blood mononuclear cells (PBMCs) from five ducklings in each group. PBMCs were planted into 96-well plates with 100μL per well after being resuspended at 1 × 10^6^ cells/mL in the full medium of DMEM containing 10% FBS. Then, incubated with 100 μL complete medium containing purified E protein antigen (20 μg/mL) for 48 h, and uninfected cells cultured only in 100 μL complete medium were used as a negative control. Subsequently, 20 μL of MTS (3-(4,5-dimethylthylthiazol-2-yl)-5-(3-carboxymethoxyphenyl)-2-(4-sulfophenyl)-2H-tetrazolium; Promega, Madison, WI, USA) was added to each well, and the cells were incubated at 37 °C in 5% CO_2_ for 4 h. The OD_490_ was then measured. The average OD_490_ value of the stimulated cells divided by the average OD_490_ value of the negative controls was used to determine the stimulation index (SI).

### 2.8. Serum Cytokine Release Analysis

To assess each group’s cytokine levels, peripheral blood supernatants were collected at 0, 1, 2, 3 and 4 weeks after the initial immunization and analyzed using commercial IL-4 and IFN- ELISA assay kits (R&D, Minneapolis, MN, USA) according to the manufacturer’s procedure.

### 2.9. Statistical Analysis

All data were collected and graphed using Prism version 5.0 software (GraphPad Software Inc., La Jolla, CA, USA), and statistical analysis was performed using SPSS 15.0 (SPSS, Chicago, IL, USA). Calculations using the sample t-test (between two groups), one-way analysis of variance and Tukey’s multiple comparison tests were used to analyze the results of the comparison of the data (between multiple groups). Means and standard deviations were calculated for all of the data, and *p*-values of less than 0.05 were judged to be statistically significant.

## 3. Results

### 3.1. Construction of Recombinant Baculovirus rBac-E

**Figure 1:**
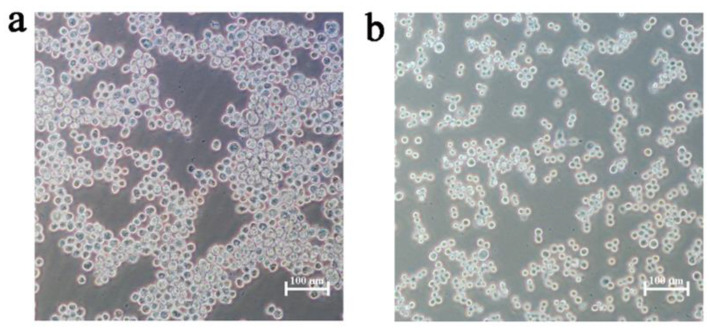
Characterization of baculovirus-infected cells. At 72 h after infection, a typical CPE was seen. (**a**) The recombinant baculovirus rBac-E was introduced into the Sf9 cells. (**b**) Cells that were mock infected with PBS. The production of bigger cells in rBac-E-infected Sf9 cells. The scale bar shows 100 μm.

Reverse transcription-PCR was used to amplify up to 1.5 kb DNA fragment (E gene) from the genome of the DTMUV strain YY5 and clone it into the pFastBac1 shuttle vector. The E gene sequence of the recombinant plasmids was confirmed to be correct by DNA sequencing. In Sf9 cells, a recombinant baculovirus known as rBac-E was constructed and propagated. As can be seen in Figure 1, Sf9 cells infected with the baculovirus gradually increased in size, with remarkable morphological changes. These typical cytopathic effects (CPEs) manifested as enlarged round cells with enlarged nuclei that filled the entire cytoplasm and poorly refractive particles in the nuclei, and loose attachment to the culture plate at 3 days post-infection. By using PCR, the recombinant baculovirus was confirmed. The recombinant baculovirus rBac-E was subcultured in Sf9 cells to the 8th generation (titer of TCID_50_ approximately was 10^7^.^17^/mL) and then stored at 4 °C for later use.

### 3.2. Recombinant Baculovirus rBac-E-Mediated Expression of E Protein in Sf-9 Cells

**Figure 2:**
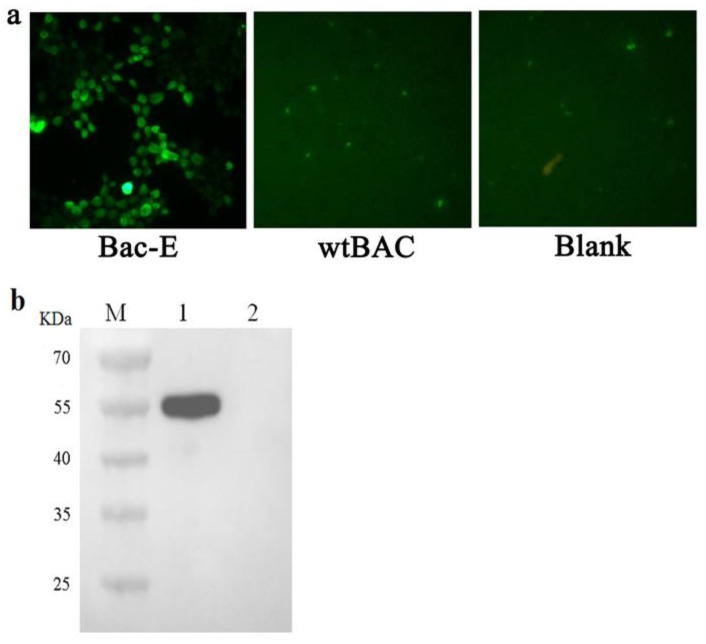
Identification of the proteins expressed in vitro by recombinant baculovirus in Sf9 cells. (**a**) Immunofluorescence analysis (IFA) of E glycoprotein protein expression in Sf9 cells infected with rBac-E. Following 48 h, cells were first using mouse anti-E polyclonal serum, followed by goat anti-mouse IgG, which was FITC-conjugated, and was then examined under a fluorescence microscope. (**b**) Western blotting study using mouse anti-E serum on lysates from cells infected with recombinant baculovirus. The rBac-E in lane 1 and Sf9 cells were infected with wtBac as a negative control in lane 2, respectively. Lane M served as the protein standard marker.

Recombinant baculovirus rBac-E was used to infect the Sf-9 cells at a multiplicity of infection (MOI) of 1 for the expression of the E protein, and wild-type baculovirus was used as a control, respectively. By using a Western blot analysis and an indirect immunofluorescence assay (IFA) 72 h after infection, it was possible to identify the expression of the E protein in SF9 cells. As shown in Figure 2a, bright fluorescence signals could be observed in the cells infected with rBac-E; however, there was no fluorescence signal detected in the cells infected with the wild-type baculovirus. Moreover, a noteworthy band with a molecular weight of about 56 kD was found by Western blot analysis, which was comparable to the size of the target protein. In the meantime, cells infected with wild-type baculovirus showed no protein band (Figure 2b). These findings showed that recombinant baculovirus rBac-E may significantly increase DTMUV E protein expression in Sf9 cells.

### 3.3. Humoral Immune Responses in Ducklings with the Recombinant Baculovirus

**Figure 3:**
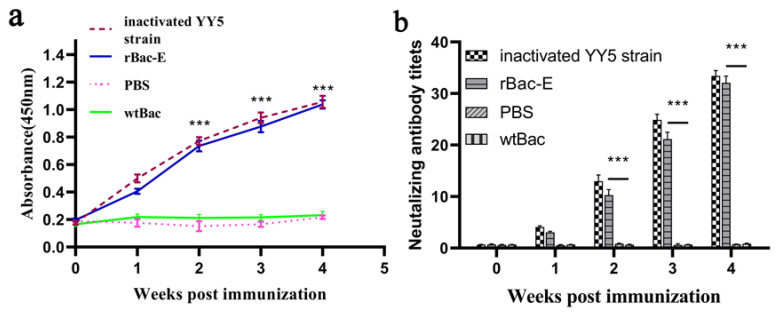
Humoral immune responses induced by recombinant baculovirus. (**a**) Anti-E glycoprotein specific antibody levels in ducklings at week 0, 1.0, 2.0, 3.0 and 4.0 after the initial immunization with rBac-E, wtBac, inactivated vaccine or PBS. Indirect ELISAs were carried out to determine the antibody titers present in the serum samples that were obtained at a variety of different times. Each dataset is presented as the mean ± SD (*n* = 10). *** *p* < 0.05, a significant difference between the rBac-E group vs. wtBac or PBS groups respectively. (**b**) Changes in serum neutralization antibody titers in different groups of ducklings after immunization with rBac-E, wtBac, inactivated vaccine and PBS, respectively. Serum samples were collected from three animals from each group randomly at weeks 0, 1.0, 2.0, 3.0 and 4.0 after the initial immunization. The endpoint titers were expressed as the reciprocal of the highest serum dilution, resulting in the neutralization of virus activity by 50%. Data are presented as mean ± SD (*n* = 3). *** *p* < 0.05, significant difference between rBac-E group vs. wtBac or PBS groups, respectively.

To investigate whether rBac-E could incite the DTMUV-specific immune response in vivo. All ducklings immunized with rBac-E developed specific antibody titers two weeks after the primary immunization, which were significantly higher than those of the Bac-wt and PBS control groups (*p* < 0.05, Figure 3a). Following booster immunization, the mean antibody level of the rBac-E group significantly increased. A high antibody level was also observed in the group vaccinated with the inactivated vaccine. There was no statistically significant difference between this group and the rBac-E group. None of the ducklings from the negative controls, wtBac and PBS, produced any detectable E-specific antibodies.

Next, neutralizing antibody titers were also determined in the same samples. As shown in Figure 3b, the neutralizing antibody titers from immunized animals with recombinant rBac-E protein and inactivated YY5 strain vaccine had a similar changing trend with the specific antibody titers, significantly higher than that of the other two control groups, the Bac-wt and PBS control groups (*p* < 0.05). Taken together, these data showed that the DTMUV E protein had good immunogenicity and effectively induced humoral immune responses.

### 3.4. Analysis of Lymphocyte Proliferation Reaction

**Figure 4:**
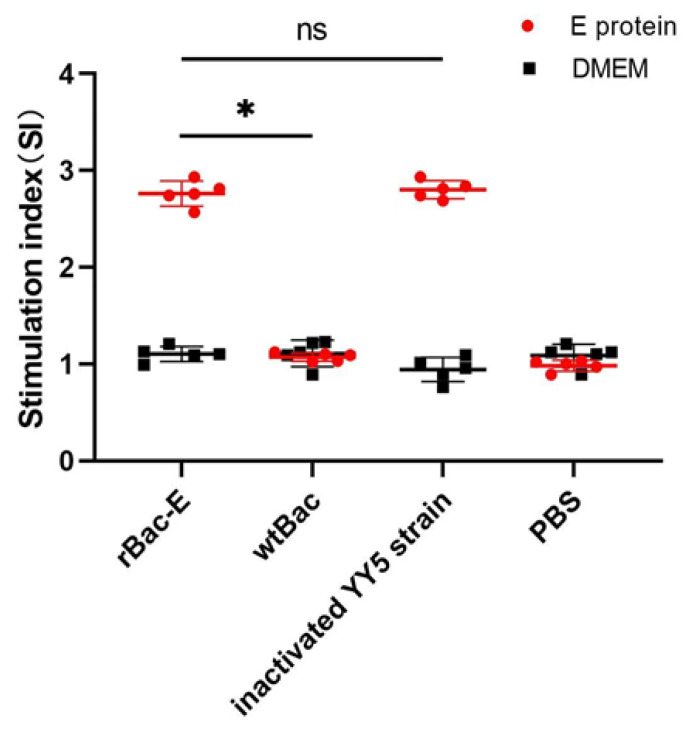
Lymphocyte proliferative responses in ducklings given various immunogens during vaccination. Data were presented as the mean concentration S.D. for the samples (*n* = 5) that were obtained at 4 weeks following primary immunization. When compared to the wtBac and PBS control groups, ducklings inoculated with rBac-E exhibited a strong lymphocyte proliferation response (* *p* < 0.05). In addition, the SI value of the rBac-E group and the inactivated vaccine group was no significant difference (^ns^ *p* > 0.05).

Four weeks following the main immunization, we evaluated the lymphocyte proliferative responses to further investigate the cellular immune responses elicited by rBac-E. The results showed that four weeks following primary immunization, ducklings inoculated with rBac-E had stimulation index (SI) values that were considerably greater than those of the two control groups (*p* < 0.05) (Figure 4). In addition, there was no significant difference between the rBac-E group and the inactivated vaccine group (^ns^ *p* > 0.05). The results indicate that recombinant DTMUV E protein can induce a significant cellular immune response in ducklings.

### 3.5. Cytokine Analysis

**Figure 5:**
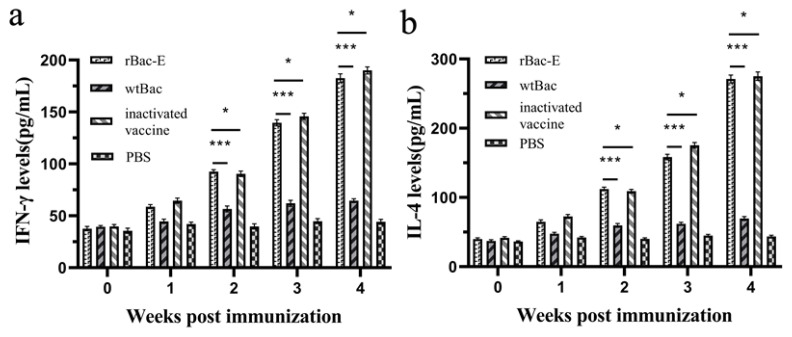
Determination of cytokine levels in ducklings after vaccination. Serum samples were collected at 0, 1.0, 2.0, 3.0 and 4.0 weeks following primary immunization for cytokine analysis using ELISA kits to measure levels of (**a**) IFN-γ and (**b**) IL-4. The results presented are shown as mean values with corresponding standard deviations. The statistical analysis revealed that the rBac-E group was stimulated with the wtBac control group with a *p*-value of <0.05 (***), while the rBac-E group was stimulated with the inactivated vaccine group with a *p*-value of >0.05 (*).

We examined the cytokine levels (IFN-γ and IL-4) in duckling serum to understand more about the cellular immune responses carried out by the DTMUV E protein. The levels of cytokine were similar in all groups before primary immunization, as shown in Figure 5. Nonetheless, it was evident that at 3.0 and 4.0 weeks following the initial immunization, the levels of IFN-γ and IL-4 in the ducklings of the rBac-E group were statistically higher than the two control groups (*p* < 0.05); however, there was no significant difference between the inactivated vaccination group and the rBac-E group (Figure 5a,b).

### 3.6. Protection of Ducklings against the DTMUV Challenge

**Figure 6:**
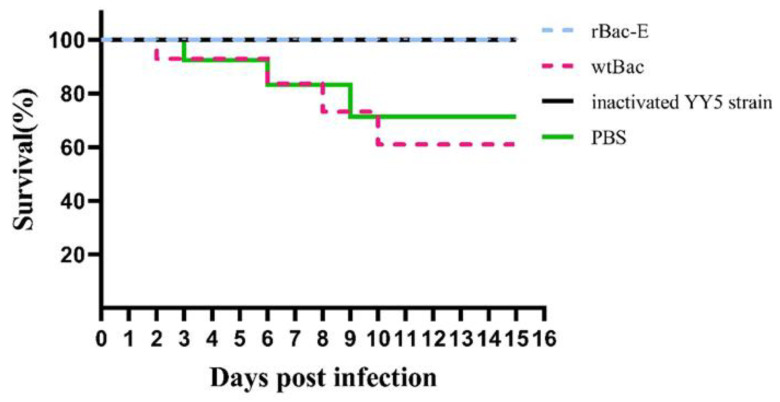
Survival curves of ducklings (*n* = 10) after challenge. Using the Kaplan–Meier approach, the statistical significance of death rate variations across groups was established and examined with a Log-rank (Mantel–Cox) test. A significant difference between the rBac-E group vs. wtBac or PBS groups, respectively.

Immunized ducklings were challenged with a dose of DTMUV strain YY5, which is lethal in 50% of recipients at 4.0 weeks after the primary immunization, to further evaluate the immune protection provided by recombinant DTMUV E protein (LD50). After the challenge, all of the ducklings were then housed in an isolation facility and observed for 14 days. The ducklings of the wtBac group and PBS group showed typical symptoms, including appetence, green-colored feces, ataxia, leg spasm and even paralysis in the later stage, and the incidence was 100%. Although 2 of 10 ducklings immunized with rBac-E were observed with very slight signs of illness, the symptoms disappeared after 10 days. All ducklings of the rBac-E group and the inactivated strain vaccine group were protected against challenges with virulent DTMUV, as shown in Figure 6. In contrast, the ducklings of the wtBac group and PBS group had survival rates of 60% and 70%, respectively.

## 4. Discussion

The process of delivering antigens plays a crucial role in triggering a protective immune response with vaccines targeting pathogens [25]. One of the benefits of using the adenoviral vector system is its capacity to produce large quantities of Pseudotyped baculovirus, deliver the foreign gene to actively dividing cells, and generate a replication-incompetent virus. These characteristics enhance the biosafety of the system [26]. Numerous vaccines have successfully utilized recombinant Pseudotyped baculovirus-expressed proteins from various infectious agents [27,28]. There are several infectious diseases, including the duck Enteritis virus (DEV), the highly pathogenic avian influenza virus, H5N1 (HPAIV H5N1), and the duck Tembusu virus (DTMUV), that pose some of the most significant challenges to the duck enterprise. Vaccinations continue to be the most economical method of eradicating these pathogens [29]. However, the H5N1 and DTMUV oil adjuvant-inactivated vaccines now on the market are typically not very immunogenic in ducks, necessitating high antigen doses and occasionally several injections [30,31]. The laying duck industry has suffered significant financial losses ever since the infection caused by the Duck Tembusu viral disease spread to China in 2010 [3,24,32]. The most efficient method of disease prevention and control is vaccine immunization. Inactivated and attenuated vaccines for DTMUV infection are already available and play a significant part in the prevention and management of DTMUV infection [18,33,34]. In a previous study, it was also proposed that glycoprotein E could be applied as a potential vaccine candidate to control DTMUV infection in young ducks [35]. However, both of the above two traditional vaccines have potential problems, such as high production cost, certain side effects, and possibly strong virulence. Therefore, the development of novel DTMUV vaccines is imperative.

The main function of humoral immunity is to generate neutralizing antibodies, which have a protective impact by rendering the virus non-infectious [36]. Neutralizing antibodies are capable of attaching themselves to specific parts of the viral surface, called antigenic determinants, thereby incapacitating the virus and preventing its ability to attach to and infect cells. This mechanism serves as a crucial defense against infection, inhibiting the occurrence and transmission of the virus [37]. Compared with traditional vaccines, recombinant subunit vaccines have significant advantages. First, subunit vaccines activate the immune response using a single component of the pathogen and do not contain pathogenic nucleic acids, which increases their safety. Second, the manufacturing process of recombinant subunit vaccines is straightforward, quick and cost-effective [38]. In addition, the immune response generated from subunit vaccines can be distinguished from wild virus infection, which is conducive to the diagnosis and control of the disease. Baculovirus has many advantages as a vaccine vector, such as relatively simple preparation and good biosafety within animals. As a result, the subunit vaccine has a wide range of potential applications in the prevention and management of infectious diseases [39,40].

For example, research on the CSFV subunit vaccine has made good progress. Bouma et al. [41] used the baculovirus–insect cell system to efficiently express the CSFV E2 protein and used it as a vaccine to protect pigs against the challenge with high doses (100 LD50) of CSFV. Similarly, Madera et al. developed a recombinant E2 vaccine based on the expression of baculovirus-insect cells. Animal experiments showed that the use of a single dose of this vaccine (KNB-E2) can produce high levels of specific and neutralizing antibodies in immunized pigs and protect them against the challenge with wild CSFV [42]. Currently, subunit vaccines based on CSFV E2 protein have been licensed for production and use in pig farms in Europe and China.

Several investigations have demonstrated that the primary target antigen of neutralizing antibodies is the flavivirus E-glycoprotein [43], and many have demonstrated that vaccines made from E-proteins can protect animals against West Nile and Japanese encephalitis virus infection [44,45]. Therefore, it should be an ideal target for the development of a DTMUV subunit vaccine. Therefore, we effectively created a recombinant baculovirus in this study that expresses the DTMUV E gene in Sf9 cells, and we further assessed its immunogenic qualities and capacity to defend against challenges. Our results showed that the recombinant E protein not only could be highly expressed in Sf9 cells but also could induce high levels of IFN-γ and IL-4 in ducklings and stimulate lymphocyte proliferation significantly higher than that in the wtBac group (*p* < 0.05). In addition, the recombinant E protein could also produce a strong humoral response, inducing the production of DTMUV-specific antibodies in immunized ducklings. Furthermore, similar to an inactivated vaccine, the recombinant E protein also exhibited 100% protection against the lethal challenge in ducklings. These findings imply that the development of DTMUV vaccines may benefit from the use of recombinant baculovirus vectors as a viable antigen-delivery strategy.

## 5. Conclusions

In conclusion, the recombinant DTMUV E protein generated by the baculovirus expression system in our study can induce an effect on humoral and cellular immune responses, which can protect ducklings against a virulent strain of DTMUV strains. These findings show that the recombinant E protein is highly immunogenic and that it can be an effective strategy for the prevention and management of DTMUV.

## Data Availability

All relevant data or analyzed during this study are included in this published article. Further inquiries can be directed to the corresponding authors.
